# RDFScape: Semantic Web meets Systems Biology

**DOI:** 10.1186/1471-2105-9-S4-S6

**Published:** 2008-04-25

**Authors:** Andrea Splendiani

**Affiliations:** 1 UPRES-EA 3888 - Laboratoire d'Informatique Médicale, Faculté de Médecine, Université Rennes 1, Rennes FR-35043, France; 2 Unité de Biologie Systemique, Institut Pasteur, Paris FR-75015, France

## Abstract

**Background:**

The recent availability of high-throughput data in molecular biology has increased the need for a formal representation of this knowledge domain. New ontologies are being developed to formalize knowledge, e.g. about the functions of proteins. As the Semantic Web is being introduced into the Life Sciences, the basis for a distributed knowledge-base that can foster biological data analysis is laid. However, there still is a dichotomy, in tools and methodologies, between the use of ontologies in biological investigation, that is, in relation to experimental observations, and their use as a knowledge-base.

**Results:**

RDFScape is a plugin that has been developed to extend a software oriented to biological analysis with support for reasoning on ontologies in the semantic web framework. We show with this plugin how the use of ontological knowledge in biological analysis can be extended through the use of inference. In particular, we present two examples relative to ontologies representing biological pathways: we demonstrate how these can be abstracted and visualized as interaction networks, and how reasoning on causal dependencies within elements of pathways can be implemented.

**Conclusions:**

The use of ontologies for the interpretation of high-throughput biological data can be improved through the use of inference. This allows the use of ontologies not only as annotations, but as a knowledge-base from which new information relevant for specific analysis can be derived.

## Background

The role of ontologies in the Life Sciences domain has increased in recent years, as the development of high-throughput measurement technologies has made it a data-intensive discipline. Ontologies are necessary for the annotation and the interpretation of large datasets, for the integration of heterogeneous information and for the creation of common languages across disciplines, ranging from the Life Sciences to Healthcare.

The success of the Gene Ontology (GO) [1] is an example of the usefulness of ontologies. GO is a unique resource for uniform annotation of gene products across organisms; and it is used to relate experimental data and knowledge on processes and functions of genes on a high-throughput scale. A typical case of this use of GO is the functional characterization of patterns of gene expression data [2].

The development of ontologies such as GO has been driven at first by the need of a wide-coverage annotation of the entities of their domain. The resulting ontologies provide a large shared terminology, but they have limited ontological commitment. Current research is also focusing on clear and formal definition of entities, relations and their properties [3][4][5][6]. As a result, bio-ontologies are shifting from almost terminological resources used for annotation of biological entities, to a formal representation of a knowledge domain that allows inference of biologically meaningful facts [7][8].

Regarding the integration of heterogeneous information, ontology development in the Life Sciences is increasingly adopting the Semantic Web [9] in particular through the OWL language [10]. In the Semantic Web vision, information resources such as biological databases can expose their data on the web in a common language (RDF [11]), together with ontologies encoding their semantics. This de facto integrated set of resources can be queried not only for its content, but also for information that is a consequence of what is explicitly asserted, given the semantics provided by ontologies. Within the same framework, languages for expressing queries such as SPARQL [12] are also defined.

An example of how heterogeneous information can be integrated and queried in this context has been developed by the W3C Healthcare and Life Sciences Interest Group [13]. It should be noted that the development of ontologies in Life Sciences covers not only the domain of biomedical entities, but also methodologies. This is the case of the Ontology for Biomedical Investigation (OBI), that aims at providing terms for the annotation of protocols, instrumentation, materials and data [14].

Focusing on biological pathways, the Pathway Commons initiative [15] readily provides access to information from resources such as KEGG [16], Reactome [17], HumanCyc [18] and others on the Semantic Web. This information is presented in a common format whose semantics is described through the BioPAX ontology [19]. Ontologies provided by Pathway Commons could be used to annotate biological entities, and their semantic annotation allows for inferences such as the abstraction of relations between entities, or the derivation of causality relations among them (this will be further discussed in the results section).

However, there is still a disconnection, in tools and methodologies, between the use of ontologies as annotations of biological entities in data analysis tools, and their use as a formal specification of entities and relations in the domain, with associated semantics. In the first case, ontologies are commonly considered as a set of semantically opaque labels associated to biological entities such as genes or proteins. These labels can be used to qualify other information about these biological entities, like experimental measurements, but relations among labels, and their semantics, are scarcely taken into account. On the other hand, tools for ontology editing such as Protégé [20] allow to edit and visualize logical constraints relating entities and properties, and to inspect the entailed consequences of their definitions, but they are unconnected from other aspects of biological investigation, such as experimental measures.

The lack of a common platform that can exploit ontologies both as an annotation tool for experimental data interpretation and as a formal representation of a domain in which new knowledge can be derived through inference has motivated the development of the work presented here. Among the tools used for data integration and analysis in the biological domain, Cytoscape is of particular interest [21]. It offers an interactive visual environment to explore biological networks whose elements can be characterized through different types of information, including experimental measurements and ontologies, by virtue of an extensible plug-in architecture.

Here we present RDFScape, a platform that integrates the ability to interpret the semantics of ontologies into a biological analysis framework such as Cytoscape.

### Brief introduction to the Semantic Web and to the terminology adopted in the present manuscript

*RDF* is a simple language that stands at the basis of the Semantic Web framework. It encodes semantics with simple statements of the form <subject predicate object>. A set of RDF statements (sometimes *facts*) can be represented as a graph.

The elements of RDF are identified by unique strings called *URI*s. Some URIs have an associated semantics. Specifically, *RDFS* and *OWL* are sets of URIs whose semantics can be used to define ontologies. Sets of related URIs can be grouped in *namespaces*.

In the present article, the term *ontology* refers both to the representation of *classes* and their relations and to their *instances*. The term *Knowledge-Base* is used almost as synonym to indicate a set of ontologies deployed in a system providing inference support.

*Inference* is the process of deriving new facts as logical consequences of known ones. These new facts are said to be *entailed* by the known ones.

## Methods

RDFScape is implemented as a Cytoscape plugin. It makes use of external libraries for managing ontologies and reasoning, and it provides additional logic to map these to graphs represented in Cytoscape.

Although its design is modular and could accommodate different libraries, the current implementation of RDFScape uses the Jena Semantic Web library [22]. This library allows for parsing of ontologies and offers a way to represent them via a graph data structure in memory (or based on a relational database engine). Inference is provided by a reasoner within the Jena Library and by Pellet [23].

RDFScape organizes data structures and inference in a peculiar way, whose usage will be presented in the results section.

Initially, a single graph data structure holds the union of all the RDF descriptions of ontologies selected by the user. Based on a selected set of standard entailments (OWL or RDFS) a second graph is then inferred. This computation is provided either by Pellet or by the Jena Inference Engine, and reasoning settings can be changed at run time. It is possible to select one of the two reasoners and, since the latter consists in different reasoning modules, the set of entailments it computes can be finely tuned.

This second graph is intended to hold an unification of the ontologies provided. Identical URIs are resolved by definition, when a formal characterization of elements in the ontologies is present, equivalence of classes, properties and individuals can be inferred in the reasoning step. When this equivalence can be inferred by other means that go beyond the semantics encoded in the ontologies, it is expected that an ontology providing explicit equivalence information is presented to the system.

The second RDF graph then provides the basis for an additional inference step that applies user-defined inference rules, implemented via the Jena backward rule engine; this results in the knowledge-base that the user can query. Details on the reasoning process in Jena can be found in [24].

Consequence of this setup is that the information entailed by user-specified rules does not affect the computation of RDFS or OWL entailments. This choice reduces the risk that user-defined rules lead to an inconsistent knowledge-base, and it is coherent with instance-rich ontologies such as BioPAX.

RDFScape maintains a connection between the data structure of the network in Cytoscape and the knowledge-base. It populates the network in Cytoscape with information as URIs, values, types for the elements (node and arcs) for which a correspondence to an ontology is found. A direct access between the internal representation of these elements and the attributes controlling their rendering is also kept. For instance, this is the case for associations between colours and namespaces. The link between these elements and the knowledge-base is based on different interfaces (SPARQL queries or direct graph access): depending on which interfaces are supported by the knowledge library in use, RDFScape provides more or less functionalities for querying and browsing the content of ontologies within Cytoscape. For instance, if the knowledge library only provides a SPARQL interface, it is not possible to retrieve information relative to the representation of a blank node (a node to which no URI is associated). On the other hand, if the knowledge library allows direct access to the RDF graph representation of ontologies, this functionality is enabled (this is the case in Jena).

RDFScape also implements I/O on files of its actual configuration parameters: these are rich data structures including ontologies, settings, inference rules, graph patterns that are grouped on the basis of work-cases called analysis contexts.

### Requirements

RDFScape requires the following software and libraries:

Cytoscape (at least version 2.4)

Jena (at least version 2.5)

Pellet (at least version 1.5)

No specific requirements are given for hardware and software: RDFScape is a Java based cross platform project whose requirements are equivalent to the fore-mentioned software.

## Results

A number of interesting synergies result from the enrichment of Cytoscape with Semantic Web technologies. As ontologies represented in the semantic web framework are networks of concepts themselves, they can be treated as graphs within Cytoscape and hence visualized (and analysed) taking advantage of its interactive features.

At the same time, ontologies can be used to annotate, and hence query elements in networks representing biological entities and experimental data.

Herein ontologies are not just seen as a set of annotations, but as a knowledge-base where the explicit representation of semantics is the basis to infer additional information. This semantics can then be extended in a user-specific way through the definition of inference rules that enrich the knowledge-base at run-time.

Here we present some of the results obtained through this integration.

### Ontology query and navigation

RDFScape provides a system for visualizing and querying ontologies represented in OWL within Cytoscape (more precisely, RDFScape targets mainly their RDF representation).

A set of features improves the readability of this visualization of ontologies as networks. For example, objects of datatype properties can be visualized as attributes of nodes representing the corresponding subjects, and visual features such as node shapes or colours can be associated to these attributes (as well as on a namespace basis). It is possible to select which resources should be visible, based on their namespaces.

Networks represented in Cytoscape can be populated in several ways: through the use of queries, through an interacting browsing system or through the visual definition of graph patterns.

In the first case, the plugin presents the user with a choice of panels to perform queries: a SPARQL query panel (where the user can enter the SPARQL query text, for which a template with the proper declaration of namespaces is provided), a class based query panel (a drop down list of classes in selected ontologies) or a string matching based query panel (a panel where the user can enter a text to be matched exactly or partially by ontology terms or their annotations).

**Figure 1:**
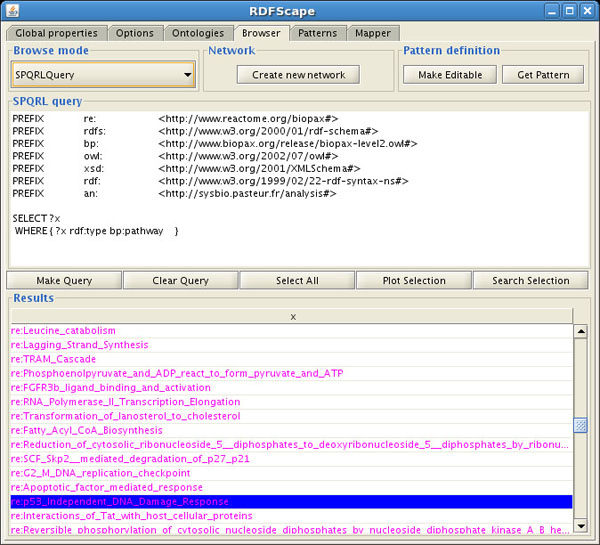
**SPARQL query panel**. The SPARQL query panel provided by RDFScape. This figure shows a simple query and the result on the Reactome Homo sapiens ontology.

After performing a query, a list of results is returned as a table (Figure 1). The user can then select a subset or all of the results and plot them into Cytoscape.

**Figure 2:**
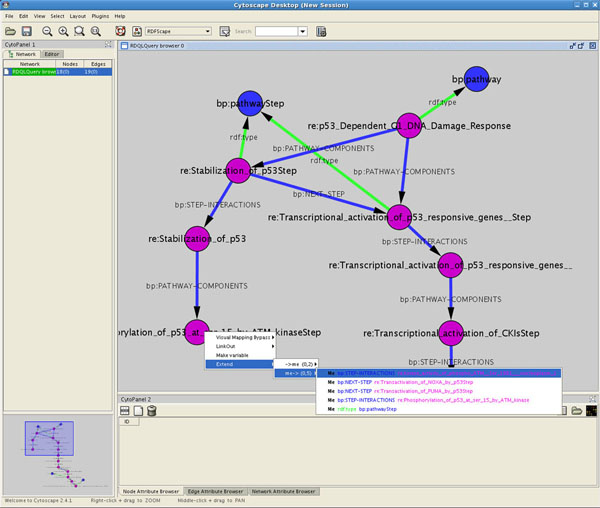
**Browsing ontologies in RDFScape**. This figure shows the contextual-menu mechanism for the interactive expansion of ontologies. The current selection highlighted in the menu will result in the addition of the statement: “re:Phosphorilation_of_p53_at_ser_15_by_ATM_kinaseStep bp:PATHWAY-STEP re:kinase_activity_of_phospho_ATM_Ser__1981___nucleoplasm_1”. Browsing ontologies at this level of details is not intuitive. Later in this paper it will be shown how, through reasoning, ontologies can be transformed to provide information easy to understand by the user.

Whenever elements visualized in Cytoscape are relative to entries in ontologies, RDFScape presents an interactive browsing system: right-selecting a node prompts a contextual menu with all the statements that have this node as object or subject. Selecting one of these menus entries leads to the addition of the relative statement to the Cytoscape network (Figure 2).

Both of these query methods only yield results within the namespaces that are selected as relevant by the user.

**Figure 3:**
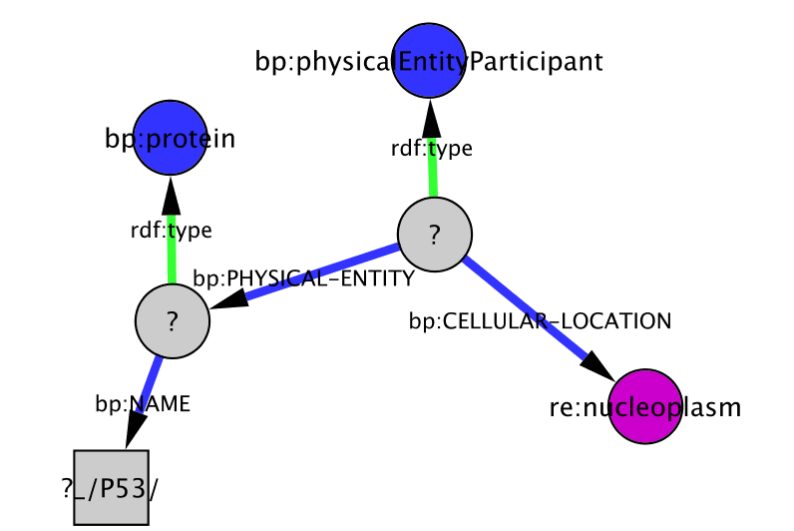
**Example of a visual query**. The pattern shown represents the query: “All the elements of type protein whose name contains the string “P53” that are part of an interaction taking place in the nucleoplasm region”. This pattern was generated while browsing the Homo sapiens Reactome export in Pathway Commons, by declaring as variable two of the elements. It presents several elements describing the structure of a pathway: these are from the BioPAX ontology (blue in the figure). The object of the CELLULAR-LOCATION property is a term defined in Reactome (in purple). Results of this query on the Reactome Homo sapiens ontology are presented in Table 1. This query can be used both to map its results to an existing Cytoscape network, or to generate a new network from all possible occurrences of the relative pattern in the ontology.

Right-selecting a node or an arc prompts also other menu items, as the possibility to declare it a variable. This feature, coupled with the possibility to select sub-networks proper of Cytoscape, allows the definition of graph-patterns or “visual queries” (An example of visual query is presented in Figure 3, the corresponding results are reported in Table 1).

**Table 1:** Results from the query in Figure 3

**Protein**	**physicalEntityParticipant**
TP53	re:p53_ser_15_phosphorylated__nucleoplasm
TP53	re:p53_protein__nucleoplasm_
TP53BP1	re:_53BP1_nucleoplasm_1
TP53BP1	re:_53BP1__nucleoplasm

This table shows results from the query of Figure 3 on the Reactome Homo sapiens ontology. For Proteins, objects of the NAME property are presented instead of their URIs. PhysicalEntityParticipant is an “utilityClass” in BioPAX: it represents the state or context of an element in an interaction, not a biological entity.

**Figure 4:**
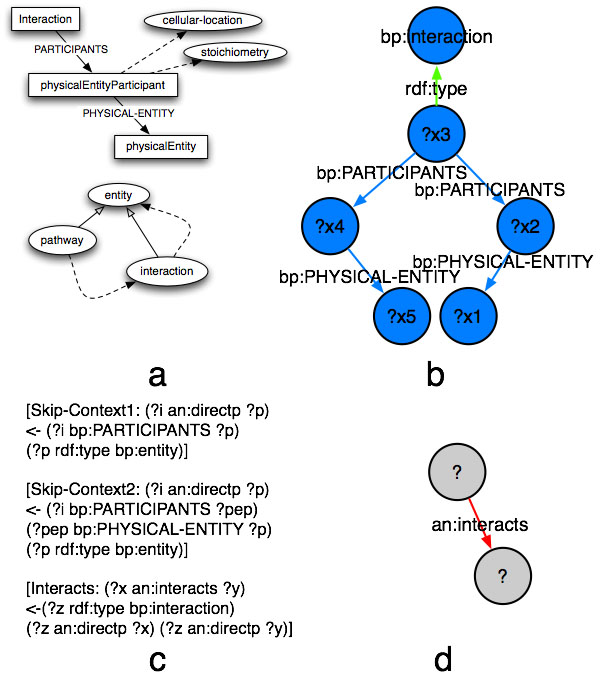
**Abstraction of interactions from pathways**. A) A fragment of BioPAX (entity, pathways, interactions) and the particular representation of interactions through physical entity participants. B) A visual query corresponding to two interacting elements. C) A set of axioms that allows abstraction of interactions and simplification of their representation. Note that new assertions inferred through these axioms are identified by a new namespace (here “an” in short). D) A visual query on the resulting intuitive representation.

**Figure 5:**
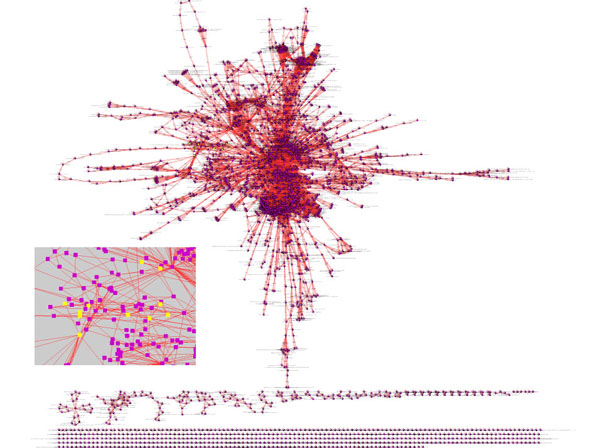
**Abstraction of Reactome Homo sapiens pathways as an interaction network**. In the small detail picture, elements in yellow are returned from a query of all elements interacting with RNAs (a total of 30 elements matching this criterion was found).

These visual queries can be saved in libraries and they can be used both to map their results to an existing Cytoscape network, or to populate a new network (an example of the latter usage is given later in Figure 5, resulting for the query in Figure 4d).

It is important to note that unlike other biological oriented software, queries on ontologies in RDFScape are not only relative to biological entities and their annotation. They can also target relations among elements and more general patterns that can be defined on the RDF representation of an ontology (that is, both nodes and arcs can be variables).

Finally, queries can lead to the selection of results on a graph representing an ontology, or to the selection of a set of nodes in a graph representing a biological network (for instance, a protein-protein interaction network), once the latter is linked to one or more ontologies.

### Support for inference on ontologies

The target of queries in RDFScape is not only the knowledge stated in ontologies, but also the knowledge that can be inferred from them. The inference procedure is customizable in two distinct ways, that we have shortly introduced in the methods section. First, some options are available to perform a subset of all inferences proper to the OWL/RDF semantics. There is a trade-off between the coverage of possible entailments computed and the execution time, and in many cases only a subset of possible inferences is relevant. It is thus possible to tune the system to maximize performance in specific cases. One very common example is the use of a reasoner to efficiently compute only transitive closures when the ontologies considered are taxonomies.

Second, in addition to the knowledge that can be derived through this standard inference, a set of rules specified by the user is processed for the production of additional statements. These rules are simple production rules matching a graph pattern on the left side.

Two facts should be noted on the use of reasoning in RDFScape.

First, custom inference rules can be saved in libraries and applied at run time. That is, the interpretation of the facts in ontologies can be interactively varied by the user.

Second, additional logic to interpret ontologies can be provided in two ways, via the aforementioned inference rules, or via additional ontologies to be added to the knowledge-base. The latter approach is analogous to the one that was used in [8].

These two ways overlap in their expressiveness, but none of them is exhaustive. However, It may be argued that additional logic encoded in ontologies would not be necessary if those ontologies were properly formalized to begin with.

### An example: abstracting pathway ontologies to interaction data

We show how the features of RDFScape, including inference, can be used to provide a semantic-based transformation of a knowledge-base through the following use case:

“Visualize a set of pathways as an interaction network”.

As a first step we consider a subset of Pathway Commons, in particular a subset of Reactome, represented in BioPAX (level 2). This ontology provides classes and relations for the description of biological pathways. For instance it defines classes such as “Catalysis”, “Control”, “Interaction”, that are related by the “subclassOf” relation (every instance of Catalysis is an instance of Control, and every instance of Control is an instance of Interaction).

An introduction to the BioPAX ontology is out of the scope of this work, however we refer to Figure 4 to introduce some simple concepts used here.

A fragment of the class hierarchy in BioPAX is shown in Figure 4a, as well as the particular way in which interactions are represented. Interactions are classes. Elements that participate in an interaction (or any of its subclasses) are related to it through an additional class “Physical Entity Participant”. This has the meaning of “the physical element with the features it has when participating in a specific interaction”, thus representing context information. Pathways are, for the scope of the present work, collections of interactions.

In order to derive an interaction network from a set of pathways represented in BioPAX, we need to perform two operations.

First, we need to abstract as “interactions” all of its subclasses. For instance when querying a network for interacting elements, those that are annotated as participating in a Biochemical Reaction should be also retrieved. This can be achieved through the computation of standard OWL entailments. In particular, the subset of OWL that can be expressed with RDFS is sufficient.

The representation of an interaction, at this point, would still not be intuitive: a query for an interaction would be represented as in Figure 4b.

We would like to “view” this interaction network as a graph representing biological entities as nodes, and interactions as edges. A set of custom inference rules (Figure 4c) allows for this transformation. The result of these rules is the assertion of a new property “interacts” between nodes participating in the same interaction (as inferred from the previous reasoning step).

A visual query yielding all interactions known in an ontology will now look like in Figure 4d.

We apply this transformation to the Homo sapiens release of Reactome in Pathway Commons. This knowledge-base is directly accessible on the web. However, access to its content is on a single pathway basis. We therefore downloaded the entire version of Reactome in Pathway Commons, and loaded it from a local file.

No interactions are recovered from a SPARQL query unless inference is activated. When RDFS inference is activated, 3323 interactions are retrieved.

We apply RDFS inference and the set of custom rules introduced earlier. Then we extract from the ontology only the “interactions” we have now defined. The result of the application of this procedure to the entire ontology is shown in Figure 5. Here we show also the application of a query for all proteins interacting with RNAs. In this case the result of a query appears as a selection of nodes within the overall interaction network.

Although not shown here, it would be possible to integrate this information with other data such as microarray data: size of nodes representing RNAs could for instance be made proportional to the fold change of RNA presence in a stimulus-response experiment.

It should be noted that the derivation of an interaction network from a set of pathways was realized without a custom representation of data. The knowledge-base used was available and in principle directly accessed on the web (Pathway Commons): we only provided definitions for our interpretation of BioPAX elements.

### Towards reasoning on pathways

Here we show how inference can be used on pathways to answer specific queries. We refer to [25] for an introduction to queries on pathways and we focus on an example derived from this work: “Find all genes whose expression is directly or indirectly affected by a given compound”.

In order to keep this presentation simple, and in order to evaluate it on the Pathway Commons knowledge-base, we consider a related simpler query:

“Find all compounds whose expression is directly or indirectly affected by a given compound”. This query is similar in the form of reasoning required to answer the former query, but it allows to define easily a meaning for “affects”: focusing on biochemical reactions, “a compound A affects a compound B if it can be used directly or indirectly in its synthesis”.

**Figure 6:**
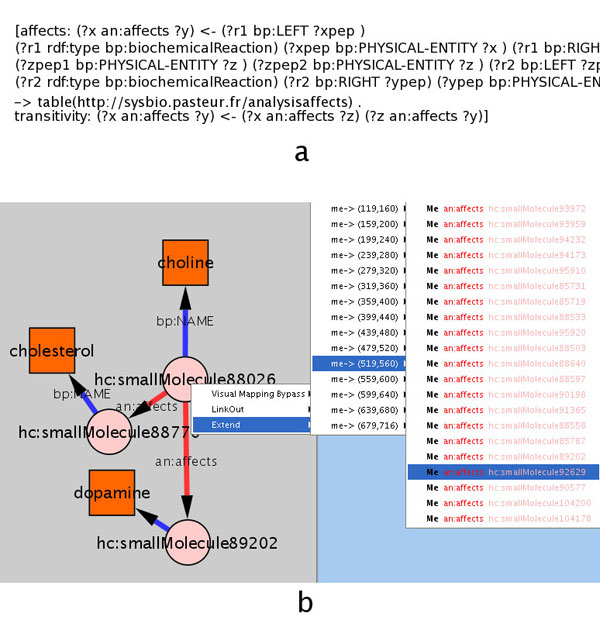
**Reasoning on pathways.** A) Rules defining a possible “affects” relations between compounds in biochemical reactions. B) Interactively browsing HumanCyc through the inferred relation. The relation currently selected is smallMolecule88206 (Choline) affects smallMolecule92629 (Adrenaline).

This can be stated through the set of axioms illustrated in Figure 6a. Note that we have expressed the transitivity of the property “affects” through production rules. Another alternative would be to declare it transitive within OWL (which would have resulted in a more efficient use of the reasoner).

Figure 6b shows an example of interactive browsing of the HumanCyc ontology following this new property.

### Availability

RDFScape is released under a LGPL license and is available at [26]. It is a prototype: the intended audience of this project are researchers interested in exploring how semantic web technologies can be applied in biological investigation.

## Discussion

Since the goal of the semantic web is to enable the web to become a distributed knowledge-base, in principle it would be possible to point RDFScape to a set of URLs of ontologies of interest, to define an interpretation for these, and to derive information that can enrich the analysis performed within Cytoscape.

We illustrated this approach in two examples with ontologies relative to biological pathways, but it can be extended to a heterogeneous domain [13]. In fact, a consistent amount of information is being published in the semantic-web framework, including resources as Pathway Commons, all the OBO ontologies [27] and Uniprot [28].

This vision is still constrained by current ontologies and tools. The use (and re-use) of URIs is at the basis of the semantic web framework. Up to now, different ontologies often refer to the same entities with different URIs. This requires a URI resolution step between loading ontologies and reasoning on the resulting knowledge base (we have avoided this step in the examples by focusing on single ontologies). Furthermore, most of the bio-ontologies currently available scarcely adopt OWL expressivity. The examples that we have presented rely only on their RDF description (the same results would have been obtained by using a RDFS reasoner).

This limits the extent to which, when dealing with different ontologies, a reasoner is able to determine relations among their classes.

The tools that support the semantic web also present relevant limitations. Most notably, current reasoners require that all the information that they process is present in memory, which poses a scalability problem.

These limitations in the implementation of the semantic web are proper of the early stages of adoption of a new technology. Despite these limitations, we have shown how ontologies and reasoners in the semantic web framework can already be used for real tasks.

This has required some knowledge of details of the representation of ontologies and of the reasoning process that is unlikely to be held by a researcher whose area of interest is primarily in the Life Sciences. In order to improve the usability of RDFScape we have addressed this issue by providing “analysis contexts” where ontologies, reasoning settings and rules can be prepared for the user as an expandable base. The two examples we presented are defined in two analysis contexts, and users can abstract pathways ontologies as interaction networks or inspect causal relations in them without needing to know the internals of the BioPAX ontology.

In the contribution of the semantic web to Life Sciences, RDFScape fills a gap in the availability of tools that rely on ontologies for biological data analysis, since no other tool presents the ability to use a reasoner on a standard knowledge base within an open environment such as Cytoscape.

We trace a comparison between RDFScape and other related tools, that are intended to represent prototypical examples of their classes.

We chose the Cytoscape BioPAX loader [29] and GOlorize [30] as examples of other Cytoscape plugins processing ontologies, PathwayTools [31][32] as a relevant representative of tools for pathway analysis, BioDASH [33] as an example of projects that provide a “semantic visualization” of ontologies (in this case based on the standard Fresnel [34] dictionary) and Ontoviz [35], OWLViz [36], Jambalaya [37] as ontology visualization tools (these are Protégé plugins).

In this comparison, we consider four features: (1) whether a tool is based on semantic web technologies, (2) whether it allows reasoning, (3) whether it provides an interactive visual environment and (4) whether it supports the integration of ontologies and experimental data. Results of this comparison are shown in Table 2.

**Table 2:** Comparison of related tools with RDFScape

	**Semantic web based**	**Reasoning**	**Interactive environment**	**Integration of ontologies and experimental data**
**RDFSCape**	yes	yes	yes	yes
**BioPAX loader**	limited (BioPAX only)	no	Yes	yes
**GOlorize**	No (but uses OBO ontologies)	No	Yes (advanced layout depending on ontological information)	Yes
**PathwayTools**	No	Yes	Yes, advanced	Yes
**BioDASH**	Yes	Limited	Limited	Yes
**Ontoviz/OWLViz**	Yes	Limited	Yes	No
**Jambalaya**	Yes	Limited	Yes	No

RDFScape presents a unique combination of features.

Ontoviz, OWLViz and Jambalaya allow interactive visualization of OWL ontologies (in RDFScape visualization is limited to the RDF level), but they don't have the ability to relate them to other biological information. The Cytoscape BioPAX loader addresses the XML representation of BioPAX and lacks support for inference. GOlorize provides an interesting ontology-driven layout feature, but its scope is limited to rendering and layout tasks. Pathway Tools provides a rich set of tools but is not based on semantic web technologies (it must be noted however that Pathway Tools is only a part of a pathway project that includes BioCyc and its owl representation). The scope of BioDASH is similar to that of RDFScape, but with focus on the integration of heterogeneous ontologies rather than on reasoning.

The ultimate goal of RDFScape is to improve the use of ontologies in systems biology investigation. In particular it addresses the problem of associating biological functions, as defined in ontologies, to high-throughput observations of biological systems.

Cytoscape provides a platform to visualize and analyse data relative to an actual biological system in specific conditions. The semantic web provides a distributed knowledge base on what is known on this biological system as a potential system. RDFScape provides the link between the two.

It therefore realizes an intelligent annotation system where the object of queries on the potential properties of a given biological subject is not only what is explicitly asserted in ontologies, but also what can be inferred from them.

An example of its usage would be to derive an interaction network from microarray data and compare it to the networks induced by the “interacts” or “affects” relations introduced in the previous examples.

Given the plugin architecture of Cytoscape, and its flexible use of reasoning, RDFScape can address a significant range of data in the systems biology domain and provide relevant interpretations.

Future developments of RDFScape will target scoring functions to determine which parts of the ontological knowledge on a subject are related to given experimental observations.

### Notes on performance

Performance of RDFScape is strictly related to those of the Cytoscape rendering system, the libraries used to manage ontologies and the reasoner selected. It is highly variable and influenced by many factors, beside the size of ontologies: the constructs used by these, the settings of the reasoner and the inference rules defined by the user. In queries, the lack of the optimizer in the underlying libraries results in variability of performance depending on their formulation.

Here we provide some empirical measurement of performance, with a breakdown in times needed to load and parse ontologies, compute inference, execute queries, map results to Cytoscape, layout and render the resulting network. We consider the previously introduced task of abstracting pathway ontologies to interaction networks, and the Homo sapiens release of Reactome. This ontology contains approximatively 27000 individuals grouped in 40 classes. Its RDF/XML representation is about 27Mb in size. Execution times are measured on a standard Desktop Machine (Intel Core 2 Duo 2GHz, 2GB of RAM, Java VM v. 1.5) and are reported in Table 3.

**Table 3:** Results of the empirical evaluation of RDFSCape performance

**Task**	**Time (sec)**
Loading and parsing (38M, 260k statements)	10-20 (a)
OWL reasoning through Pellet, custom inference rules through Jena / query: retrieve all interactions (~19k results)	60/12 (b)
OWL (partial) reasoning through Jena, custom inference rules through Jena / query: retrieve all interactions (~19k results)	5/5 (b)
Plotting results into Cytoscape (~19k relations)	30
Graph rendering in Cytoscape	7

(a) minimum and maximum time measured (variability reflects the usage of different disk subsystems).

(b) reasoning time and query time are reported separately.

All times reported are the approximate average of two measurements.

Since some of the reasoners used by RDFScape perform part of the reasoning process on demand, it is not always possible to account for reasoning independently of queries. We have provided two grouped measures relative to different settings.

These results show that it is possible to use inference within an interactive environment: query times are contained and the most demanding task, reasoning, is still performed in an acceptable time (this task must be performed only when reasoning parameters or the set of ontologies of interest change).

However, it should be noted that wrong settings of the inference process, and possibly some ontologies, can easily result in unacceptable reasoning and answering times, in execution of infinite loops by the reasoner, or can make this exceed the memory capacity of an average workstation.

## Conclusions

We have developed RDFScape, a plugin for Cytoscape that enables it to use ontologies represented in the semantic web framework.

In RDFScape, it is possible to query and visualize not only the information explicitly asserted in ontologies, but also what can be inferred from them, where the inference process can be tuned by the user within Cytoscape to produce her own interpretation of data. Beside that, RDFScape enables new queries functionalities in Cytoscape, like SPARQL queries, visual queries or interactive browsing of ontologies.

The introduction of reasoning in a platform oriented to biological data analysis fills a gap in the availability of semantic web tools in the Life Sciences area.

We have shown how RDFScape can enhance visualization and understanding of pathway ontologies in two example cases: abstracting pathway information as interaction networks, and deriving causal information between elements.

Future development of RDFScape will target the link between ontologies and experimental data.

## List of abbreviations used

GO, Gene Ontology, OWL Ontology Web Language, RDF Resource Description Framework, SPARQL, Simple Protocol And RDF Query Language, W3C World Wide Web Consortium, OBI Ontology for Biomedical Investigation, KEGG, Kyoto Encyclopaedia of Genes and Genomes, URI, Uniform Resource Identifier, RDFS, Resource Description Framework Schema, RNA, Ribonucleic Acid, LGPL, Lesser General Public License, URL, Uniform Resource Locator, OBO, Open Biomedical Ontology, GB Gigabyte, GHz Gigahertz, RAM, Random Access Memory, XML, eXtensible Markup Language, EU, European Union, VM, Virtual Machine.

## Competing interests

The authors declare that they have no competing interests.

## Authors' contributions

The author conceived RDFScape, implemented it and developed the examples presented in this manuscript.
